# 10-Gingerol Suppresses Osteoclastogenesis in RAW264.7 Cells and Zebrafish Osteoporotic Scales

**DOI:** 10.3389/fcell.2021.588093

**Published:** 2021-03-03

**Authors:** Liqing Zang, Kazuhiro Kagotani, Hiroko Nakayama, Jacky Bhagat, Yuki Fujimoto, Akihito Hayashi, Ryoji Sono, Hirotaka Katsuzaki, Norihiro Nishimura, Yasuhito Shimada

**Affiliations:** ^1^Graduate School of Regional Innovation Studies, Mie University, Tsu, Japan; ^2^Zebrafish Drug Screening Center, Mie University, Tsu, Japan; ^3^Tsuji Health & Beauty Science Laboratory, Mie University, Tsu, Japan; ^4^Tsuji Oil Mills Co., Ltd., Matsusaka, Japan; ^5^Department of Life Sciences, Graduate School of Bioresources, Mie University, Tsu, Japan; ^6^Department of Integrative Pharmacology, Graduate School of Medicine, Mie University, Tsu, Japan; ^7^Department of Bioinformatics, Advanced Science Research Promotion Center, Mie University, Tsu, Japan

**Keywords:** bone resorption, osteopenia, bone regeneration, *Danio rerio*, natural product

## Abstract

Osteoporosis is the most common aging-associated bone disease and is caused by hyperactivation of osteoclastic activity. We previously reported that the hexane extract of ginger rhizome [ginger hexane extract (GHE)] could suppress receptor activator of nuclear factor kappa-B ligand (RANKL)-induced osteoclastogenesis in RAW264.7 cells. However, the anti-osteoclastic components in GHE have not yet been identified. In this study, we separated GHE into several fractions using silica gel column chromatography and evaluated their effects on osteoclastogenesis using a RAW264.7 cell osteoclast differentiation assay (*in vitro*) and the zebrafish scale model of osteoporosis (*in vivo*). We identified that the fractions containing 10-gingerol suppressed osteoclastogenesis in RAW264.7 cells detected by tartrate-resistant acid phosphatase (TRAP) staining. In zebrafish, GHE and 10-gingerol suppressed osteoclastogenesis in prednisolone-induced osteoporosis regenerated scales to promote normal regeneration. Gene expression analysis revealed that 10-gingerol suppressed osteoclast markers in RAW264.7 cells [osteoclast-associated immunoglobulin-like receptor, dendrocyte-expressed seven transmembrane protein, and matrix metallopeptidase-9 (*Mmp9*)] and zebrafish scales [osteoclast-specific cathepsin K (CTSK), *mmp2*, and *mmp9*]. Interestingly, nuclear factor of activated T-cells cytoplasmic 1, a master transcription regulator of osteoclast differentiation upstream of the osteoclastic activators, was downregulated in zebrafish scales but showed no alteration in RAW264.7 cells. In addition, 10-gingerol inhibited CTSK activity under cell-free conditions. This is the first study, to our knowledge, that has found that 10-gingerol in GHE could suppress osteoclastic activity in both *in vitro* and *in vivo* conditions.

## Introduction

Osteoporosis is a progressive metabolic disease in which the bone structure deteriorates due to a decrease in bone density associated with aging and lifestyle factors. Fractures are a typical symptom, with a higher prevalence in women than men and an estimated 200 million patients affected worldwide (Svedbom et al., 2013). Bone homeostasis is maintained through a balance between osteoclast bone resorption and formation. The imbalance of bone resorption and formation caused by malnutrition, lack of exercise, and reductions in estrogen and testosterone leads to decreased bone density and osteoporosis (Karsenty and Wagner, 2002; Boyle et al., 2003; Suzuki and Yoshida, 2010).

The pharmacological treatment of osteoporosis is to inhibit osteoclast activities or decrease the number of osteoclasts present to promote bone formation. Bisphosphonates, a class of chemicals that are most commonly used to treat osteoporosis, are adsorbed onto bone surfaces and taken up by osteoclasts to induce apoptosis and promote bone formation (Drake et al., 2008; Rogers et al., 2011). In addition, bisphosphonates directly inhibited receptor activator of nuclear factor kappa-B ligand (RANKL)-stimulated osteoclast differentiation and fusion in RAW264.7 cells (Abe et al., 2012). While bisphosphonates are generally well-tolerated drugs clinically, several adverse effects, such as gastroesophageal irritation and osteonecrosis of the jaw, have been reported (Kennel and Drake, 2009). Instead, natural products containing essential nutrients for bone metabolisms, such as calcium (Heaney, 2006), vitamin D3 (Ooms et al., 1995), vitamin K (Palacios, 2006), and other bioactive compounds, are expected to prevent osteoporosis in elderly people with few side effects compared to conventional drugs (Shen et al., 2009; An et al., 2016; Ohyama et al., 2018; Wang et al., 2018). For example, soybean isoflavone acts as a female hormone analog in maintaining bone metabolism in postmenopausal women (Wei et al., 2012), and citrus β-cryptoxanthin promote bone formation and suppresses osteoclastogenesis in tissue cultures (Yamaguchi and Uchiyama, 2004).

Ginger, the rhizome of the plant *Zingiber officinale*, is used throughout the world as a common cooking spice. It has also been used as a natural medicine with several health-promoting properties being identified, including gastrointestinal regulation (Yamahara et al., 1990) and anti-emetic and anti-inflammatory functions (Dugasani et al., 2010). We previously reported that a hexane extract prepared from ginger [ginger hexane extract (GHE)] suppressed osteoclastogenesis in RANKL-induced RAW264.7 cells (Ito et al., 2016). However, because GHE is composed of many components, we were unable to identify the compounds inhibiting osteoclastogenesis.

Zebrafish are attractive animal models for developmental biology and human disease studies because of their numerous advantageous features (Dooley and Zon, 2000; Meunier, 2012). Recently, the zebrafish scale model system has emerged as a model for bone research (Pasqualetti et al., 2012). The body surface of zebrafish is covered with dermal skeleton elasmoid scales, which have a similar structure and function to the mammalian lamellar bone (Sire et al., 1997). Both osteoblasts and osteoclasts have been detected in adult zebrafish scales, and their interaction regulates bone formation and remodeling similar to those observed in human bones (Bergen et al., 2019; Kobayashi-Sun et al., 2020). Zebrafish scales are easy to harvest, and new scales can be regenerated within a few days, providing a powerful tool to observe the mechanisms of bone remodeling (de Vrieze et al., 2011). An osteoporosis model zebrafish has been reported to show osteoporosis-like phenotypes in regenerating zebrafish scales undergoing prednisolone treatment (de Vrieze et al., 2014). In addition, Pasqualetti et al. (2015) reported that treatment with the bisphosphonate alendronate (AL) rescued the osteoporotic phenotype induced by prednisolone using adult zebrafish scales, which further underlined the utility of using zebrafish scales as a model for anti-osteoporotic drug discovery.

In this study, we searched for active constituents in GHE that suppress osteoclastogenesis using RAW264.7 cells and zebrafish scales. As a result, we discovered 10-gingerol as a new compound that inhibits osteoclastogenesis.

## Materials and Methods

### Ethics Statement

All animal procedures were performed according to the Japanese Animal Welfare Regulatory Practice Act on Welfare and Management of Animals (Ministry of Environment of Japan), in compliance with international guidelines.

### Sample Preparation

Ginger hexane extract was prepared from the residue after squeezing the juice from the raw ginger rhizome, as previously reported (Ito et al., 2016). In brief, *n*-hexane was added to the residue, and extraction was carried out below 10°C. After filtration, GHE was obtained by removing the *n*-hexane under reduced pressure. The yield ratio of GHE from the raw ginger was approximately 0.1–0.5%.

For administration to zebrafish, emulsions of GHE and 10-gingerol (Chem Faces, Wuhan, China) were prepared using lecithin (PC70; Tsuji Oil Mills, Mie, Japan) according to our previous study (Kagotani et al., 2020) with slight modifications. The epithet PC70 indicates that phosphatidylcholine accounts for more than 70% of the total lecithin. Then, 5% (w/w) of PC70 (equal to 50 mg/mL) was dissolved in 70% glycerol at 60°C. A further 1% (w/w) GHE (equal to 10 mg/mL) or 0.1% (w/w) 10-gingerol (equal to 1 mg/mL) was added to the mixture, which was then completely dissolved at 60°C. Preliminary emulsification of the mixture was performed at 60°C for 5 min using a disperser (Polytron Homogenizer PT2100, Central Scientific Commerce, Tokyo, Japan). The emulsion was then treated twice at 100 MPa using an NM2-L200 emulsifying machine (Yoshida Kikai, Aichi, Japan). The resulting emulsions used in this study contained 5% PC70 with 1% GHE or 0.1% 10-gingerol.

#### Silica Gel Chromatography

Each GHE fraction was obtained using a silica gel column with an elution solvent. Briefly, 35 g of Wakogel C-300 (Fujifilm Wako Pure Chemicals, Osaka, Japan) was packed in a Φ20 × 300 mm glass column (HARIO Science, Tokyo, Japan) with *n*-hexane, and 2 g of GHE was applied to the top of the silica gel layer. GHE was sequentially treated with hexane, acetone, and ethanol and collected as a fraction containing the eluted components at a volume of 30–200 mL. Subsequently, preliminary thin-layer chromatography (TLC) analysis was performed to confirm the components contained in each fraction, and fractionation was performed to finally obtain 10 fractions. Afterward, each fraction was removed with elution solvent using an evaporator, and the yield of the fraction was determined.

#### High-Performance Liquid Chromatography Analysis

Analysis of the compounds of GHE fractions was conducted via high-performance liquid chromatography (HPLC) on an HPLC analysis system (Hitachi, Tokyo, Japan) under the following conditions: C18 column (Φ4.6 × 250 mm; Fujifilm Wako Pure Chemicals); Ultraviolet (UV) detector at 228 nm; column temperature of 40°C; mobile phase of A = H_2_O and B = CH_3_CN; gradient: 0–20 min, 30–90% B; 20–45 min 90% B; and flow rate 0.6 mL/min. 6-gingerol, 6-shogaol, 8-gingerol, 10-gingerol (Chem Faces, Wuhan, China), neral, geranial (Fujifilm Wako Pure Chemicals), phellandrene (Tokyo Chemical Industry, Tokyo, Japan), and farnecene (Sigma-Aldrich, MO, United States) were purchased as standard, respectively.

### Liquid Chromatography-Mass Spectrometry (LC/MS) Analysis

Identification was carried out on an LCMS-2010 EV (Shimadzu, Kyoto, Japan) equipped with a binary pump (LC-20AD), column oven (CTO-20A), photo-diode array detector (PDA SPD-10A-VP), and TSKgel ODS-100V (2 mm i.d. × 75 mm, 3 μm) (Tosoh Corporation, Japan). Mobile phase A was water and mobile phase B was methanol. The gradient elution was used. At 0–10 min = 10–100% B at a flow rate of 0.2 mL/min. The column temperature was maintained at 40°C. The mass spectrometric analysis was performed in the positive and negative ion mode under the conditions as follows: flow rate of the nebulizer gas (nitrogen) of 1.5 mL/min; CDL (curved desolvation line) temperature of 250°C; block heater temperature of 200°C; detector voltage of 1.5 kV; and mass scanning range of m/z 50–1000.

### TLC Analysis

The samples (each 2.5 μL), (1) 1.5% disodium prednisolone 21-phosphate/75% ethanol, (2) 2% 10-gingerol/ethanol, and (3) mixture solution of (1) and (2) (1:1 [v/v]) were spotted onto a TLC plate (5 cm × 10 cm; TLC Silica gel 60; Merck KGaA, Germany), and the TLC plate was developed with *n*-hexane:diethyl ether = 2:3 (v/v). The spots on the TLC plate were detected using UV light and then visualized as dehydrated carbide by spraying 2% Ce(SO_4_)_2_/2N H*2*SO*4* (Fujifilm Wako Pure Chemicals, Osaka, Japan) and heating in an oven.

#### Cell Culture, Sample Treatments, and Osteoclast Differentiation

RAW264.7 cells (The European Collection of Authenticated Cell Cultures, Wiltshire, United Kingdom) were seeded (3 × 10^3^ cells/cm^2^) and cultured for 24 h in α-minimum essential medium (Nacalai Tesque, Tokyo, Japan) supplemented with 10% fetal bovine serum (Biowest SAS, Nuaille, France) and 1% penicillin-streptomycin (Fujifilm Wako Pure Chemicals) at 37°C in a humidified 5% CO_2_ atmosphere. Then, the cells were treated with GHE, each GHE fraction, or 10-gingerol. After 24 h, defined as day 0, the medium was changed to osteoclast differentiation medium containing 100 ng/mL of soluble receptor activator of NF-κB ligand (sRANKL; Oriental Yeast, Tokyo, Japan) with the test compounds. The medium was replaced with fresh medium every 2 days until day 6.

### Zebrafish Experiment

Zebrafish AB strain (The Zebrafish International Resource Centre, Eugene, OR, United States) was maintained at 28°C with a standard light/dark cycle of 14/10 h in our facility according to standard operational guidelines (Westerfield, 2007). The fish were fed GEMMA Micro 75, 150, and 300 (Skretting, Fontaine-lès-Vervins, France) based on their developmental stage and length. Prednisolone and AL, purchased from Fujifilm Wako Pure Chemicals, were dissolved with water to create a 100- and 50-mM stock solution, respectively (Pasqualetti et al., 2015). Adult male zebrafish (4–6-month-old) of similar body weights and lengths were used in this study. On day 0, the fish were anesthetized in 0.05% 2-phenoxyethanol (Fujifilm Wako Pure Chemicals), and the scales on the left side of the fish (from the caudal fin to the middle of the trunk) were removed and discarded. Fish were recovered in distilled water containing 1 μg/mL of kanamycin (Fujifilm Wako Pure Chemicals) for 2 h. Scale-removed zebrafish were randomly assigned to five groups with five fish per 2 L tank. Prednisolone (PN: 25 μM), AL (10 μg/mL), 10-gingerol (0.1 μg/mL), and GHE (10 μg/mL) were administrated through the direct immersion of fish into 1.5 L of distilled water. We also administered PC70-lecithin (vehicle for 10-gingerol and GHE) to have the same amount of vehicle concentration in all experimental groups. One-third of the water for the fish was changed daily. The fish were starved during treatment to accelerate the induction of osteoporosis induction (Park et al., 2016). On day 8, the regenerated scales were harvested from each individual under anaesthetization within the area where the scales were removed on day 0.

#### Tartrate-Resistant Acid Phosphatase Staining

For RAW264.7 cells, the differentiated cells (day 6) were fixed with 4% formaldehyde solution in phosphate buffered saline (4% PFA; Pharma, Tokyo, Japan) for 10 min at room temperature, washed with purified water, and stained with tartrate-resistant acid phosphatase (TRAP) staining solution (Cosmo Bio, Tokyo, Japan), according to the manufacturer’s protocol. The stained cells were imaged using a microscope (CKX41; Olympus, Tokyo, Japan) with an attached camera (DP21; Olympus) or a BZX-710 fluorescent microscope (Keyence, Tokyo, Japan). Analysis of the suppression of osteoclastogenesis was carried out by counting the number of multinucleated cells or a quantification method using ImageJ (Fiji distribution, version 1.52p, National Institute of Health, Bethesda, MD, United States). Mature osteoclasts were defined as having nuclei of more than 11 per cell (Ito et al., 2016; Zarei et al., 2016).

For zebrafish, the harvested scales were fixed in 4% [PFA PFA (Pharma)] for 1 day at 4°C. The scales were subsequently stained with TRAP staining solution (TRAP/ALP Stain Kit; Fujifilm Wako Pure Chemicals) according to the manufacturer’s protocol. The TRAP-positive intensities of the stained scales were quantified using ImageJ software. To avoid the effect of the different sizes of each scale, all values were normalized to the scales’ area (de Vrieze et al., 2014).

### Pit Formation Assay

Pit assay to measure bone resorptive activity was conducted using Bone Resorption Assay Kit 24 (Cosmo Bio, Tokyo, Japan) according to the manufacturer’s protocol. RAW264.7 cells were seeded in fluoresceinamine-labeled chondroitin sulfate (FACS) and calcium phosphate-coated 24-well plates (1 × 10^4^ cells/well) containing phenol red-free α-minimum essential medium supplemented with 10% fetal bovine serum and 1% penicillin-streptomycin. One day after seeding, the cells were treated with or without 10-gingerol. On day 2, osteoclastogenesis was induced by adding sRANKL (100 ng/mL) as described above. The medium was refreshed every 2 days. Six days after sRANKL treatment, 100 μL of the medium and 50 μL of the resorption assay buffer were transferred into the wells of a 96-well plate and the released FACS was quantified using a Victor2 multilabel plate reader (485/535 nm; PerkinElmer, Boston, MA, United States). After washing the cells with 5% sodium hypochlorite, the resorbed areas on the plate were visualized using a BZX-710 fluorescence microscope (Keyence).

#### Bone Matrix Vital Staining

Bone matrix formation at the scales was evaluated by exposing the live fish to successive pulses of vital dyes, as previously described with a minor modification (Pasqualetti et al., 2015). Briefly, zebrafish were treated with PN, 10-gingerol, GHE, and AL, as previously described. On day 6, the fish were vitally stained with 0.005% Alizarin red S (pH adjusted to 7.0–7.5; PG Research, Tokyo, Japan) overnight in the dark. The next morning, fish were rinsed thrice for 10 min in the system water. A second pulse was performed on the evening of day 7 using a 0.005% calcein solution (pH adjusted to 7.0–7.5; Dojindo Laboratories, Kumamoto, Japan) overnight in the dark. At the end of the treatment, after repeated washes with system water, the regenerated scales were removed and fixed with 4% PFA overnight at 4°C. Fluorescence images were observed and taken using a BZ-X710 fluorescence microscope (Keyence, Tokyo, Japan).

#### Quantitative Reverse Transcription PCR (qPCR)

For RAW264.7 cells, total RNA was extracted using RNeasy mini (Qiagen, Hilden, Germany). For zebrafish, the harvested scales from each individual were gathered and homogenized in 1 mL TRIzol Reagent (Invitrogen, Carlsbad, CA, United States). Total RNA was isolated using TRIzol reagent combined with the RNA cleanup protocol using RNeasy Mini-prep Kit (Qiagen) (Zang et al., 2014). cDNA synthesis from 500 ng (RAW264.7 cells) or 200 ng (zebrafish scales) total RNA was performed using a ReverTra Ace qPCR RT Kit (Toyobo, Osaka, Japan).

qPCR was performed in cDNA samples using a Power SYBR Green MasterMix (Applied Biosystems, Foster City, CA, United States) and the ABI StepOne Plus Real-Time PCR System (Applied Biosystems). Each qPCR analysis was performed in triplicate on three or four independent biological samples. The sequences of the primers used in this study are shown in Supplementary Table 1. For zebrafish, three housekeeping genes *actin beta 1 (actb1*), glyceraldehyde-3-phosphate dehydrogenase (*gapdh*), and *18S* ribosomal RNA, were validated, and *actb1* was found to be the most stable and was, thus, selected as the internal standard to determine the relative values of target mRNA expression. The relative mRNA expression levels in RAW264.7 cells were determined using endogenous standards of mouse *Gapdh*.

### Fluorescent Immunohistochemistry

RAW264.7 cells were cultured in an eight-well slide and chamber (Watson, Tokyo, Japan) and osteoclast differentiation was induced as described above, with or without 10-gingerol (2.5 μM). On day 3 after the commencement of differentiation, the cells were fixed with 4% [PFA PFA (Pharma)]. Fluorescent immunohistochemistry (FIHC) was carried out as described elsewhere. Antibodies against the nuclear factor of activated T-cells cytoplasmic 1 (NFATc1; Santa Cruz Biotechnology, Santa Cruz, CA, United States) and Tumor necrosis factor (TNF) receptor-associated factor 6 (Traf6; Santa Cruz Biotechnology) were used at 1:100, and a secondary antibody against mouse IgG (Alexa Fluor 594 conjugated; Cell Signaling Technology, Danvers, MA, Unites States) was used at 1:1000. ProLong Gold Antifade Reagent with 4′,6-diamidino-2-phenylindole DAPI (Thermo Fisher Scientific, Waltham, MA, United States) was used for nuclear staining. The fluorescent images were captured using a BZX-710 fluorescent microscope (Keyence).

### Cathepsin K Inhibition Assay

The activity of 10-gingerol and GHE on Cathepsin K (CTSK) inhibition was evaluated using the CTSK Inhibitor Screening Kit (Fluorometric) (BioVision, Mountain View, CA, United States) according to the manufacturer’s protocol. Briefly, stock solutions of 10-gingerol (10 mM) and GHE (10 mg/mL) were diluted ten times with the reaction buffer. Next, 10 μL of the diluted compounds was added to 50 μL of the reaction buffer containing 50 ng of human CTSK enzyme and pre-incubated for 10 min. Finally, 40 μL of the reaction buffer containing 2 μL CTSK substrate (Ac-LR-AFC; 10 mM) was added to monitor the AFC fluorescence cleaved by CTSK. Fluorescence was measured using a Wallac ARVO^TM^ SX 1420 Multilabel Counter (Perkin Elmer, Waltham, MA, United States) at an excitation wavelength of 405 nm with emission monitored at 535 nm. As an inhibitor control, FF-FMK (10 μM; BioVision) was used according to the manufacturer’s protocol. The relative inhibition rate of each sample was then calculated. Each compound was subjected to triplicate measurements.

### Statistics

The data were analyzed statistically using Student’s *t*-test or one-way analysis of variance with the Bonferroni-Dunn multiple comparison procedure, depending on the number of comparisons to be performed, using GraphPad Prism version 8 (GraphPad Software, San Diego, CA, United States). A *p*-value of less than 0.05 denoted the presence of a statistically significant difference between treatments.

## Results

### Ginger Hexane Extract Fractions Containing Gingerols Suppress Osteoclastogenesis

**FIGURE 1:**
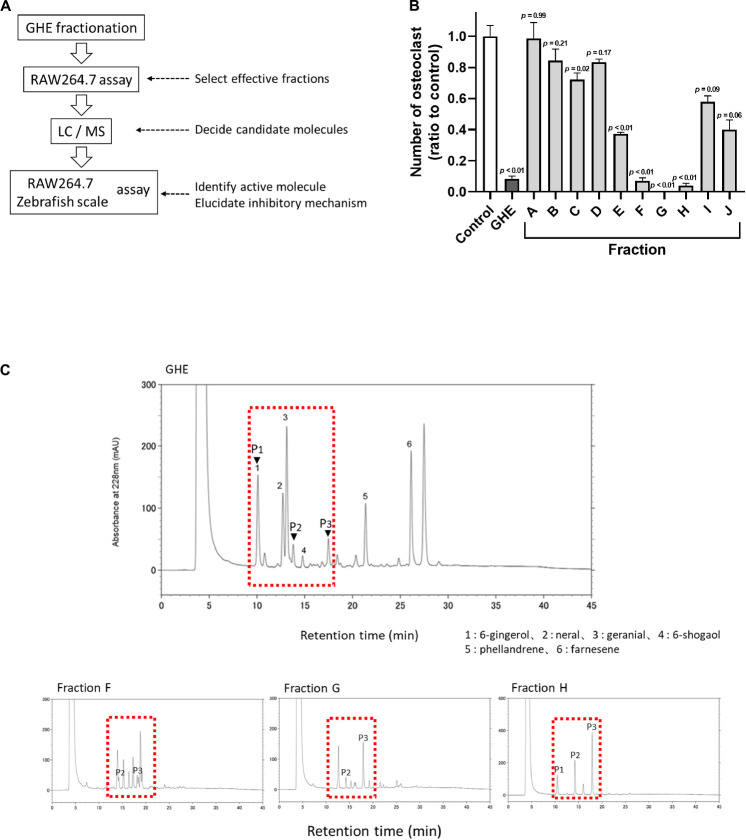
Ginger hexane extract (GHE) fractions containing gingerols suppress osteoclastogenesis in RAW264.7 cells. **(A)** Experimental design to identify anti-osteoclastic constituents in GHE. **(B)** The number of TRAP-positive multinucleated osteoclasts containing more than 11 nuclei. *n* = 3, error bars indicate SE; *p* values are calculated compared to control. The representative TRAP stained images for each fraction are shown in Supplementary Figure 1. **(C)** Chromatograms of HPLC analysis of GHE and Fractions F, G, and H. The peaks were detected at UV 228 nm absorbance. The peak numbers 1, 2, 3, 4, 5, and 6 refer to 6-gingerol, neral, citral, 6-shogaol, phellandrene, and farnesene, respectively. Identification of P1, P2, and P3 indicated by arrowheads was performed by LC/MS analysis.

The overall strategy to identify anti-osteoclastic compounds in GHE is represented in Figure 1A. To identify the responsible compounds in GHE, we first prepared its fractions separated by silica-gel column chromatography and performed the RAW264.7 osteoclast differentiation assay. The elution solvent and yield of each obtained fraction are described in Table 1. As shown in Figure 1B, fractions F, G, and H strongly suppressed the numbers of multinucleated mature osteoclasts (*p* < 0.01) compared to the control, similar to GHE (*p* < 0.01). TRAP staining can be used to detect osteoclasts as TRAP is a well-known marker of osteoclast resorption in bone metabolism. Representative images of TRAP-stained RAW264.7 cells with GHE or each fraction are depicted in Supplementary Figure 1. HPLC analysis identified some similar peaks (P1–P3) shown in the dotted box in fractions F, G, and H (Figure 1C). The retention times of P1, P2, and P3 were 10.25, 13.78, and 17.65 min, respectively.

**TABLE 1:** The amount of each GHE fraction separated from different elution solvents.

Fraction	Elution solvent	The amount of extracts: mg (%)
A	*n*-Hexane = 100	490 (24.5)
B	*n*-Hexane = 100	184 (9.2)
C	*n*-Hexane : Acetone = 98 : 2 (v/v)	386 (19.3)
D	*n*-Hexane : Acetone = 93 : 7 (v/v)	322 (16.1)
E	*n*-Hexane : Acetone = 93 : 7 (v/v)	48 (2.4)
F	*n*-Hexane : Acetone = 93 : 7 (v/v)	154 (7.7)
G	*n*-Hexane : Acetone = 88 : 12 (v/v)	140 (7.0)
H	*n*-Hexane : Acetone = 88 : 12 (v/v)	178 (8.9)
I	*n*-Hexane : Acetone = 88–85 : 12–15 (v/v)	348 (17.4)
J	Acetone : Ethanol = 50–0 : 50–100 (v/v)	136 (6.8)

*The total processed amount of GHE was 2 g.*

Peaks corresponding to P1, P2, and P3 were confirmed by PDA chromatography, and information regarding these key ion peaks was obtained by LC/MS analysis. The main ion peaks of P1, P2, and P3 were detected in positive ion mode at m/z 317, 345, and 373, and in negative ion mode at 293, 321, and 349, respectively. Finally, P1, P2, and P3 were determined to be identical to standard reagents of 6-gingerol (MW = 294), 8-gingerol (MW = 322), and 10-gingerol (MW = 350), respectively, as previously reported as ginger constituents (Tao et al., 2009). Fractions A-C (no inhibitory effect on osteoclastogenesis) contained hydrocarbon compounds (farnesene and phellandrene) and aldehyde compounds (neral and geranial).

### 10-Gingerol Suppresses Osteoclastogenesis in RAW264.7 Cells and Zebrafish Regenerated Scales

**FIGURE 2:**
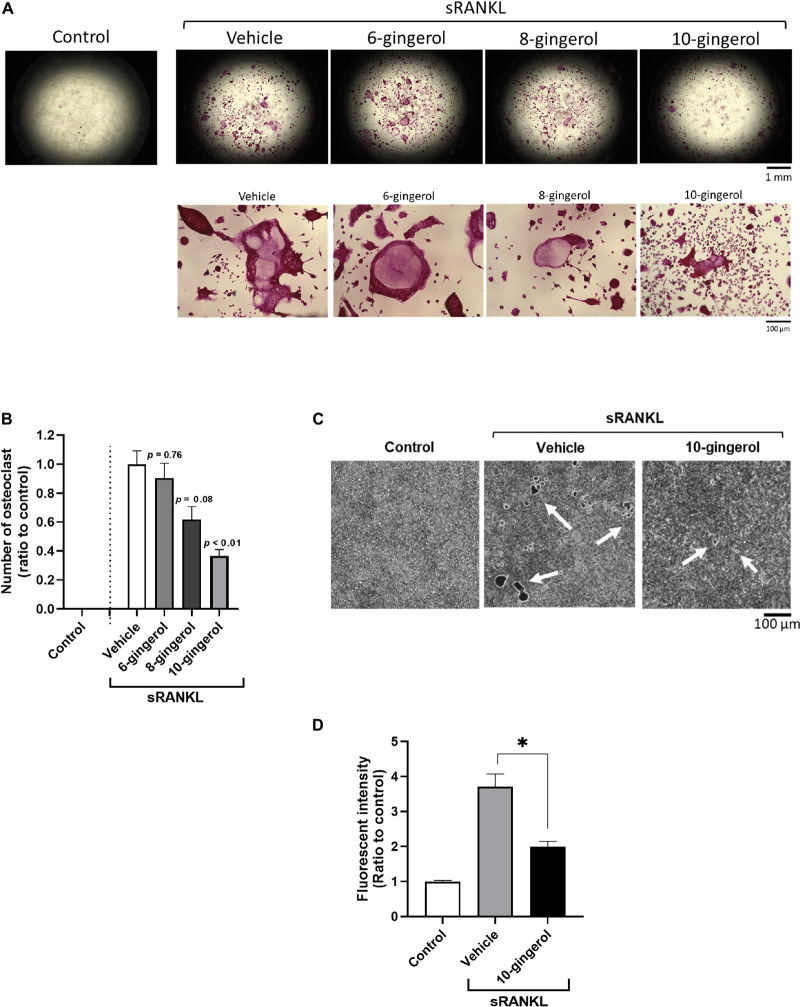
10-gingerol suppresses osteoclastogenesis in RAW264.7 cells. **(A)** Representative images of sRANKL-treated RAW264.7 cells with 6-, 8-, or 10-gingerol. Each treatment was carried out at 2.5 μM. Red indicates the TRAP-stained area. The lower panels show the magnified images of sRANKL-treated RAW264.7 cells with or without gingerols. **(B)** Quantitative analysis of TRAP-positive multinucleated osteoclasts of (A). *n* = 8, error bars indicate SE; *p* values are calculated compared to vehicle with sRANKL. **(C)** Representative images for the pit formation assay with or without 10-gingerol. White indicates pits. **(D)** Bone resorption activities of panel **(C)** were quantified by the released FACS from the well bottom. *n* = 3, error bars indicate SE; ^∗^*p* < 0.05 vs. vehicle with sRANKL.

To identify which gingerol suppresses osteoclastogenesis, we performed the RAW264.7 osteoclast differentiation assay again. We treated the cells with each gingerol (final 2.5 μM) from day 1 (1 day before the start differentiation) and performed TRAP staining on day 6. As shown in Figures 2A,B, 10-gingerol significantly (*p* < 0.01) suppressed osteoclast differentiation (red color indicated TRAP-positive area) compared to vehicle (sRANKL alone). In addition to the RAW264.7 cells, we also performed an osteoclastogenesis assay using rat bone marrow-derived osteoclast precursors. As shown in Supplementary Figure 2,10-gingerol also reduced the number of osteoclasts in TRAP staining. As some gingerols have been reported to possess anticancer effects (Lee et al., 2008; Chen et al., 2009; Ryu and Chung, 2015), we performed a cell viability assay and did not detect a significant reduction of RAW264.7 cells in all gingerols at concentrations up to 10 μM (Supplementary Figure 3). To confirm the effect of 10-gingerol on bone resorption, we performed a pit formation assay on well plates coated with FACS and calcium phosphate (CaP). The FACS bound to CaP was released from the CaP layer into the medium by osteoclastic resorption. Thus, bone resorption activity is proportional to the fluorescence intensity of FACS in the medium. On day 6 of sRANKL treatment, 10-gingerol treatment reduced the size of osteoclast bone sorption pits (Figure 2C) and also reduced the release of CaP from the bottom of the well to the medium (Figure 2D).

**FIGURE 3:**
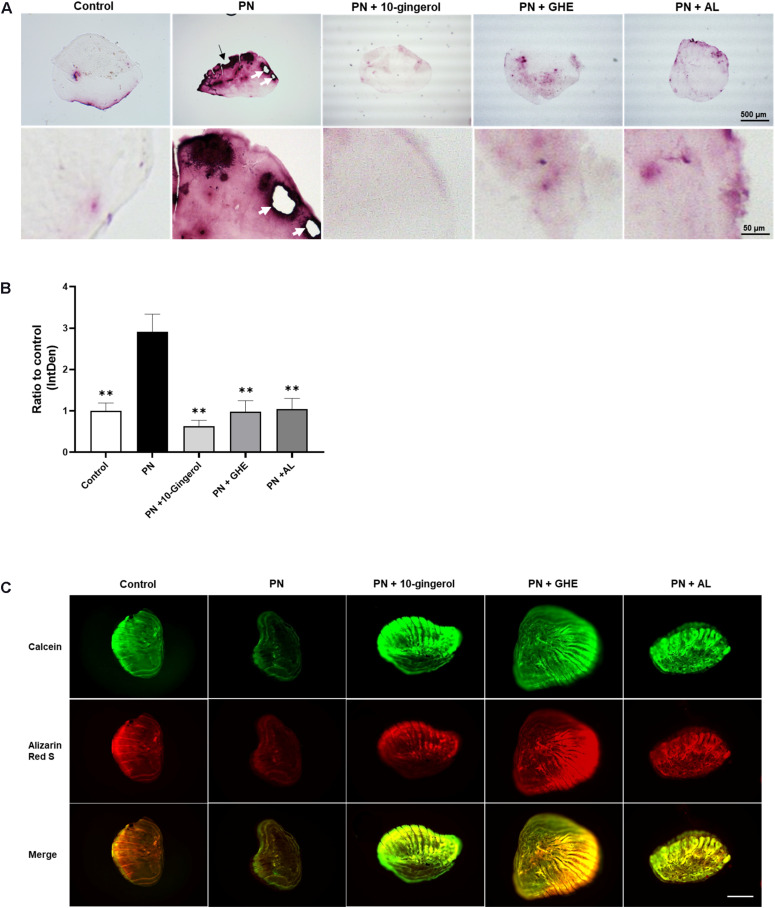
10-gingerol suppresses osteoclastogenesis in zebrafish osteoclastic scales. **(A)** Representative images for prednisolone (PN) treated regenerating zebrafish scales with 10-gingerol, GHE, or alendronate (AL; positive control). The lower panels show magnified images of the right lateral regions of the upper scales. Red color indicates the TRAP-positive area. The dark area in PN indicates the concentration of TRAP-positive cells. The black arrow indicates the resorbed edges, and white arrows show the resorption pits. For the experimental design of the zebrafish study, please see Supplementary Figure 2. **(B)** Quantitative analysis of **(A)**. Integrative densities of the red areas were measured using ImageJ. *n* = 15–20, error bars indicate SE; ^∗∗^*p* < 0.01 vs. PN. **(C)** Scales after calcein and alizarin red S staining. The upper panels show calcein staining, Alizarin red staining, and merged images, respectively. Scale bar = 500 μm.

The zebrafish scale provides a powerful tool for bone research because of its high functional similarity with human bone and easy handling. The regenerated scales can be harvested within a week, and osteogenesis can be observed, including osteoblast differentiation, matrix deposition, and mineralization (Metz et al., 2012). In addition, one of the glucocorticoids, PN, has been demonstrated to enhance osteoclast activity and matrix resorption in regenerated zebrafish scales (de Vrieze et al., 2014). To investigate the *in vivo* efficacy of 10-gingerol, we conducted a zebrafish study that induced the osteoporosis-like phenotypes by prednisolone. The experimental design for the zebrafish study is presented in Supplementary Figure 4. After the removal of scales at day 0, five adult fish were exposed to 25 μM PN alone or combined with 0.1 μg/mL 10-gingerol, 10 μg/mL GHE, or 10 μg/mL bisphosphonate AL [as the positive control (Pasqualetti et al., 2015)]. To improve transdermal delivery (Ito et al., 2016), we prepared a lecithin-based emulsion of 10-gingerol or GHE because they are highly hydrophobic. On day 8, the regenerated scales of each group were harvested, and TRAP staining was performed to visualize osteoclasts. As shown in Figure 3A, the regenerated control scales exhibited mild TRAP activity (red color as osteoclast population) with a normal round shape, whereas the PN-treated scales had an irregular shape, resorbed edges, resorption pits, and considerably increased levels of osteoclasts present, as previously reported (de Vrieze et al., 2014). 10-gingerol or GHE treatment resulted in a normal shape, few resorption sites, and decreased osteoclasts compared to PN-treated scales, as well as AL treatment. Quantitative analysis for the TRAP signal revealed significant (*p* < 0.01) decreases in the TRAP-positive osteoclasts in the scales treated with 10-gingerol (0.22-fold), GHE (0.34-fold), and AL (0.36-fold) compared to the PN-treated group (Figure 3B). We further investigated bone matrix formation using Alizarin Red S and calcein vital staining. Alizarin Red S is a red fluorescent dye that stains bone calcified matrix, and calcein is a green fluorescent chromophore that specifically binds to calcium (Mariotti et al., 2015). PN markedly decreased the scale mineralized matrix staining with both vital bone dyes compared to the results of the control scale, while 10-gingerol, GHE, and AL-treated scales exhibited strong expression of the two bone dyes, which means that the treatments rescued the loss of the mineralized matrix (Figure 3C).

To evaluate the possibility of direct inhibition of 10-gingerol on PN activity, TLC analysis was performed. As shown in Supplementary Figure 5, the spots of PN and 10-gingerol were detected on the plate, and their Rf values were 0 and 0.38, respectively. No extra spots were detected in the PN and 10-gingerol mixture solution, indicating that 10-gingerol could not bind PN directly to inhibit PN activity.

### 10-Gingerol Decreased Osteoclast Differentiation Markers *in vitro* and *in vivo*

**FIGURE 4:**
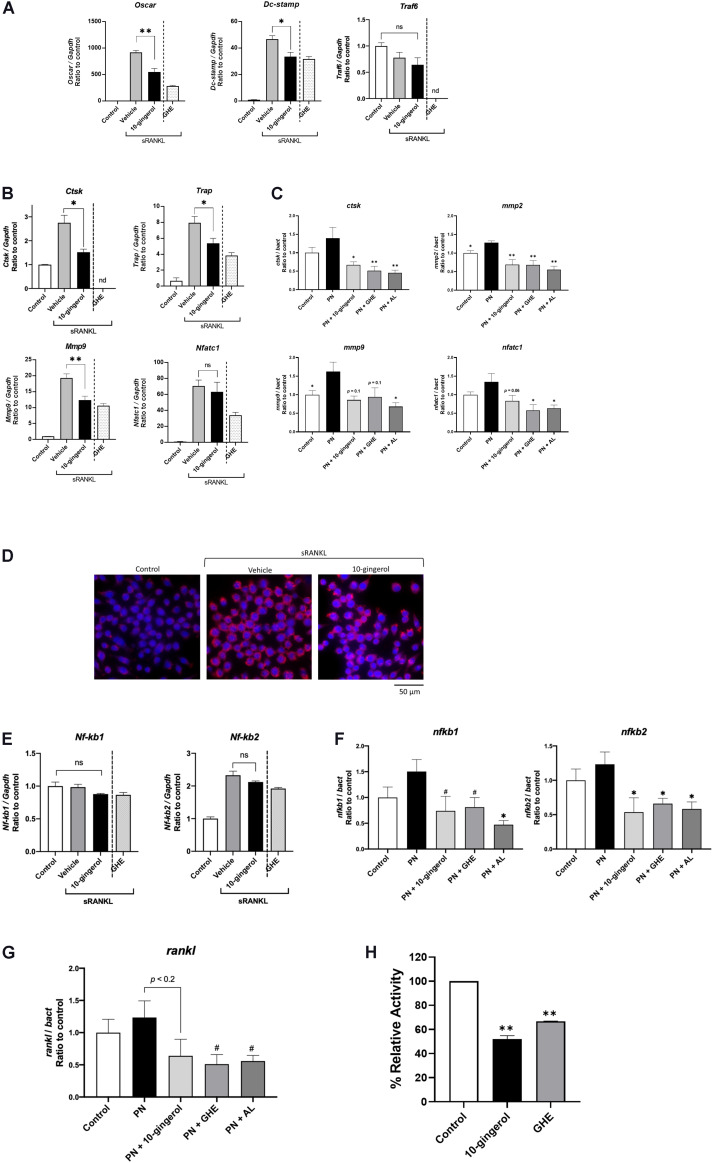
Gene expression analysis of RAW264.7 cells and zebrafish scales treated with 10-gingerol. **(A,B)** qPCR analysis of osteoclast marker genes in RAW264.7 cells with 10-gingerol. *Oscar*: Osteoclast-associated immunoglobulin-like receptor; *Dc-stamp*: Dendrocyte expressed seven transmembrane protein; *Traf6*: Tumor necrosis factor receptor-associated factor 6; *Ctsk*: Cathepsin k; Trap: Tartrate-resistant acid phosphatase; *Mmp9*: matrix metalloproteinases-9; *Nfatc1*: nuclear factor of activated T cells cytoplasmic 1. *n* = 3, error bars indicate SE; *p < 0.05, **p < 0.01. The data for GHE treatment were referred from our previous study (Ito et al., 2016). nd indicates no data. **(C)** qPCR analysis of osteoclast marker genes in osteoporotic zebrafish scales with 10-gingerol, GHE, or AL. *ctsk*: cathepsin k; *mmp2*: matrix metalloproteinases-2. *p < 0.05, **p < 0.01. *n* = 5, error bars indicate SE. **(D)** Fluorescent immunohistochemistry for *Nfatc1* in RAW264.7 cells treated with sRANKL for 3 days. Red indicates NFATc1, and blue indicates nuclei (DAPI). **(E)** qPCR analysis of *Nf-kb1* and *Nf-kb2* in RAW264.7 cells with 10-gingerol. *Nf-kb1*: Nuclear factor kappa B subunit 1; *Nf-kb2*: Nuclear factor kappa B subunit 2. *p < 0.05. *n* = 5, error bars indicate SE. **(F,G)** qPCR analysis of *nf-kb1*, *nf-kb2*, **(F)** and *rankl* **(G)** in osteoporotic zebrafish scales (PN) with 10-gingerol, GHE, or AL. *rankl*: receptor activator of nuclear factor kappa B ligand. #*p* < 0.1, *p < 0.05 vs. PN group. *n* = 5, error bars indicate SE. **(H)** Cell-free CTSK inhibitor assay of 10-gingerol (100 μM) and GHE (100 μg/mL). FF-FMK (10 μM) was used as a positive control (98.3% inhibition to the negative control). **p < 0.01 vs. negative control. *n* = 3, error bars indicate SE.

We performed qPCR analysis to validate the gene expression profiles related to osteoclastogenesis in RAW264.7 cells and zebrafish scales. In RAW264.7 cells, 10-gingerol significantly (*p* < 0.05) downregulated osteoclast differentiation markers, osteoclast-associated immunoglobulin-like receptor (*Oscar*), dendritic cell-specific transmembrane protein (*Dc-stamp*), TNF receptor-associated factor 6 (*Traf6*), *Ctsk*, matrix metalloproteinase-9 (*Mmp9*), and *Trap* mRNA expression (Figures 4A,B), similar to the GHE treatment (Ito et al., 2016). Meanwhile, mRNA levels of *Nfatc1*, an upstream promoter of osteoclast differentiation, were not reduced by 10-gingerol (Figure 4B). In addition, the expression levels of *Traf6*, which initiate RANK signaling in RAW264.7 cells upstream osteoclast differentiation, were not affected by 10-gingerol (Figure 4A).

In zebrafish scales, qPCR analysis revealed that the relative mRNA expression levels of key osteoclast differentiation markers *ctsk* and *matrix metalloproteinases-2* (*mmp2*) were significantly (*p* < 0.05 and *p* < 0.01, respectively) downregulated upon 10-gingerol administration compared to those of the PN group. The expression of *mmp9* and *nafatc1* showed trends toward decreases in the 10-gingerol group compared to **PN** (Figure 4C; *p* = 0.1 and 0.06, respectively). These results demonstrate that 10-gingerol is a major bioactive constituent in GHE that suppresses osteoclast differentiation and ameliorates osteoporosis.

Although *Nfatc1* expression was not downregulated by 10-gingerol in RAW264.7 cells (Figure 4B), osteoclast differentiation was inhibited. Thus, we performed NFATc1 FIHC in RAW264.7 cells and found that the nuclear translocation of NFATc1 was inhibited by 10-gingerol (Figure 4D). To elucidate the mechanisms underlying the inhibition of osteoclastogenesis, we performed gene expression analysis for nuclear factor kappa B (NF-kB) genes in RAW264.7 cells and zebrafish scales. As shown in Figure 4E, 10-gingerol did not affect *Nf-kb1* and *Nf-kb2* in RAW264.7 cells, whereas it downregulated *nf-kb1* (*p* < 0.1 vs. PN group) and *nf-kb2* (*p* < 0.05 vs. PN group) in zebrafish scales (Figure 4F). In addition, endogenous *rankl* mRNA expression showed a tendency (*p* < 0.2) to be downregulated by 10-gingerol in zebrafish scales (Figure 4G).

### 10-Gingerol Inhibited Cathepsin K Activity

Cathepsin K is primarily responsible for bone matrix degradation by osteoclasts and is currently the most attractive target for treating osteoporosis (Dai et al., 2020). Because 6-shogaol, a major constituent of ginger, also suppresses osteoclastogenesis by inhibiting CTSK activity (Villalvilla et al., 2014), we tested the CTSK inhibition ability of 10-gingerol and GHE under cell-free conditions. Compared with the negative control, 10-gingerol (100 μM) and GHE (100 μg/mL) reduced CTSK activity by 48.1 and 33.2%, respectively (Figure 4H).

## Discussion

### Bioactive Components of Ginger Related to Bone Metabolism

In this study, we found for the first time, to our knowledge, that 10-gingerol has anti-osteoclastic activity in GHE. Of the many bioactive components in ginger, there have been few studies regarding 6-gingerol, one of the major pungent constituents in the raw rhizomes, and its relationship to bone metabolism and those that do exist have controversial conclusions. Hwang et al. (2018) reported that 6-gingerol inhibits osteoclast differentiation via downregulation of RANKL expression in osteoblasts. Moreover, 6-gingerol was also found to increase the gene expression of osteogenic markers, increased alkaline phosphatase activity, and enhance mineralized nodule formation in osteoblast-like MG-63 cells (Fan et al., 2015). On the contrary, Khan et al. (2012) reported that oral administration of 6-gingerol promoted osteoclastogenesis on the skeleton of adult female Balb/cByJ mice. In the present study, we did not detect osteoclastic activity of 6-gingerol. In addition to 6-gingerol, 6-shogaol, a dehydrated form of 6-gingerol produced by heating, has also been reported to affect bone metabolism (Yeh et al., 2019; Kim et al., 2020). 6-shogaol inhibits osteoclastogenesis and alveolar bone resorption with the inhibition of NFATc1 nuclear translocation (Kim et al., 2020), which is necessary to transactivate the osteoclastic genes. However, 6-shogaol was not detected in the anti-osteoclastic GHE fraction (F, G, and H), as we did not heat (dehydrate) the samples during GHE preparation. In addition, 8-gingerol, which has a similar structure to 10-gingerol, is reported to have anti-platelet aggregation, and spasmolytic and immunoregulatory activities (Lu et al., 2011), while there have been no studies regarding anti-osteoclastic activity.

### Anti-Osteoclastic Mechanism of 10-Gingerol

Dugasani et al. (2010) reported that 10-gingerol has the highest anti-inflammatory and antioxidant activities compared to other gingerols. As several antioxidants inhibit osteoclastogenesis from promoting bone formation (Domazetovic et al., 2017), this assists in explaining our results.

In the differentiated RAW264.7 cells, 10-gingerol downregulated gene expression of *Oscar*, essential for the amplification of NFATc1 during osteoclastogenesis (Negishi-Koga and Takayanagi, 2009), *Dc-stamp*, a regulator of osteoclast cell fusion (Xing et al., 2012), and *Mmp9* and *Trap*, involved in bone matrix breakdown (Sundaram et al., 2007). These results are similar to those observed in GHE in our previous study (Ito et al., 2016), except *Nfatc1* expression. Although GHE downregulated *Nfatc1* expression in RAW264.7 cells (Ito et al., 2016), 10-gingerol did not (Figure 4B). We performed NFATc1 FIHC in RAW264.7 cells and found that the nuclear translocation of *Nfatc1* was inhibited by 10-gingerol, which subsequently suppresses osteoclastogenesis in *in vitro* conditions (Figure 4D). Because *Oscar* is located upstream of osteoclastogenesis signaling cascades (Negishi-Koga and Takayanagi, 2009), we supposed that the downregulation of *Oscar* by 10-gingerol would lead to inactivate *Nfatc1* protein. Traf6, an upstream of Nfatc1 protein, was not inactivated by 10-gingerol, even though the expression of the mRNA was not affected.

In osteoporotic zebrafish scales, *ctsk*, a key osteoclast protease in bone matrix degradation, (Zenger et al., 2007) and *mmp2/9*, major players for extracellular matrix degradation during bone resorption (Tat et al., 2011; Gu et al., 2014), were downregulated by 10-gingerol administration (Figure 4C). Interestingly, *nfatc1* was also downregulated by 10-gingerol, which is a different result from that of RAW264.7 cells. The reason for this gap should be elucidated through further study, though it sometimes occurs between *in vitro* and *in vivo* studies. Even with this gap in 10-gingerol’s inhibitory mechanisms in RAW264.7 cells and in zebrafish scales, the polarization step (osteoclastic precursor to multinuclear osteoclast) would be inhibited by 10-gingerol. In addition, the effects of 10-gingerol on the NF-kB pathway, an upstream pathway for Nfat1, were different between RAW264.7 cells and zebrafish scales. 10-gingerol downregulated *nf-kb1* and *nf-kb2* expression in zebrafish scales (Figure 4F), but not in RAW264.7 cells (Figure 4E). In addition, 10-gingerol downregulated *rankl* expression in zebrafish scales (Figure 4G), which was exogenous in RAW264.7 cells. Even with the differences between zebrafish and mammals, we concluded that multiple mechanisms of inhibition would exist in anti-osteoclast effect of 10-gingerol. Of course, it is necessary to perform further studies, including clinical trials.

Another possible mechanism underlying the anti-osteoclast effect of 10-gingerol is the CTSK inhibitory property. Villalvilla et al. (2014) demonstrated that osteoarthritis treatment using ginger, partially based on its constituent 6-shogaol, inhibits CTSK activity. CTSK is a lysosomal cysteine protease secreted by activated osteoclasts, which can degrade native collagen and dissolve the organic matrix (Costa et al., 2011). In recent years, CTSK inhibitors have been developed by pharmaceutical companies as potential anti-resorptive agents (Dai et al., 2020). The pharmacological effects of CTSK inhibitors on osteoporosis have been demonstrated in zebrafish, rodent models, and humans (Stoch and Wagner, 2008; Xue et al., 2019). In this study, the cell-free CTSK enzymatic assay revealed that 10-gingerol possessed CTSK inhibition ability (Figure 4H). In addition to downregulating CTSK transcript (Figures 4B,C), 10-gingerol strongly inhibited CTSK under *in vivo* and *in vitro* conditions. These findings are most likely involved in the improvement of bone resorption in the pit formation assay (Figure 2C) and osteoporotic zebrafish scales (Figure 3) through treatment with 10-gingerol. Taken together, 10-gingerol may be a novel anti-resorptive agent for osteoporosis treatment.

In the present study, we found that 10-gingerol in ginger suppressed osteoclastogenesis by improving osteoclastic symptoms in regenerated zebrafish scales. 10-gingerol should be further tested in rodent models of osteoporosis and in clinical trials because of the low toxicity derived from the natural product as a food ingredient.

## Data Availability Statement

The original contributions presented in the study are included in the article/Supplementary Material, further inquiries can be directed to the corresponding author/s.

## Ethics Statement

Ethical review and approval was not required for the animal study because all animal procedures were performed according to the Japanese Animal Welfare Regulatory Practice Act on Welfare and Management of Animals (Ministry of Environment of Japan), in compliance with international guidelines. Ethical approval from the local Institutional Animal Care and use committee was not sought, as this law does not mandate the protection of fish.

## Author Contributions

KK, YS, and NN contributed to the conceptualization. HK contributed to the methodology–chemistry. KK, HN, and YS contributed to the methodology–cell study. LZ and JB contributed to the methodology–zebrafish study. LZ contributed to the formal analysis. YS contributed to the investigation and supervision. YF, AH, and RS contributed to the resources. KK and LZ contributed to the writing–original draft preparation. JB and YS contributed to the writing–review and editing. NN contributed to the project administration and funding acquisition. All authors contributed to the article and approved the submitted version.

## Conflict of Interest

KK, YF, AH, and RS are employed by company Tsuji Oil Mills Co., Ltd. The remaining authors declare that the research was conducted in the absence of any commercial or financial relationships that could be construed as a potential conflict of interest.
