# Supplementary material for: 10-Gingerol Suppresses Osteoclastogenesis in RAW264.7 Cells and Zebrafish Osteoporotic Scales
Source: Front Cell Dev Biol. 2021 Mar 3;9:588093. doi: 10.3389/fcell.2021.588093 (PMC7978033; doi:10.3389/fcell.2021.588093)
Supplement: Supplementary file 1 [file Data_Sheet_1.docx]

Supplementary Material

# Supplementary Table

**Table S1.** Primer sequences for qPCR.

| **Zebrafish** |  |  |  |  |  |
| --- | --- | --- | --- | --- | --- |
| Symbol | Gene name | Accession  number | Forward primer   (5' - 3') | Reverse primer   (5' - 3') | Product size  (bp) |
| *ctsk* | cathepsin K | NM_001017778 | ATGATCTGGGCATGAACCAT | CCGAAGTGACGTATCCCAGT | 174 |
| *mmp2* | matrix metallopeptidase 2 | NM_198067 | TTGCTTCCCTGCAAACTTTTG | GAGCCACTTCTTTGTCTGTGTGA | 87 |
| *mmp9* | matrix metallopeptidase 9 | NM_213123 | AAATCTGTGTTCGTGACGTTTCCT | GCCGTAACGCTTCAGATACTCAT | 87 |
| *nfatc1* | nuclear factor of activated T cells 1 | NM_001045159 | AACCTTCCTCGTTCCCTCAA | CGCTGTTATCCTCCACCTCA | 153 |
|  |  |  |  |  |  |
| **Mouse** |  |  |  |  |  |
| Symbol | Gene name | Accession  number | Forward primer   (5' - 3') | Reverse primer   (5' - 3') | Product size  (bp) |
| *Oscar* | Osteoclast-associated receptor | NM_001290377 | CTGGAAGAAGTGACTCCGGC | GAGCTGATCCGTTACCAGCAG | 123 |
| *Dc-stamp* | Dendrocyte expressed seven transmembrane protein | NM_029422 | TACGTGGAGAGAAGCAAGGAA | ACACTGAGACGTGGTTTAGGAAT | 100 |
| *Ctsk* | Cathepsin K | NM_007802 | ATGTGAACCATGCAGTGTTGGTG | ATGCCGCAGGCGTTGTTCTTATTC | 119 |
| *Mmp2** | Matrix metallopeptidase 2 | NM_008610 | CACACCAACACTGGGACCTG | AGAATGTGGCCACCAGCAAG | 148 |
| *Mmp2** | Matrix metallopeptidase 2 | NM_008610 | CACACCAACACTGGGACCTG | GTA AAC AAG GCT TCA TGG GGG | 105 |
| *Mmp9* | Matrix metallopeptidase 9 | NM_013599 | GTCCAGACCAAGGGTACAGC | ATACAGCGGGTACATGAGCG | 107 |
| *Trap* | Tartrate-resistant acid phosphatase | NM_007388 | CCGCCTCTTCCCAACTCG | CATGAATCCATCTTGGCGGTG | 103 |
| *Nfatc1* | Nuclear factor of activated T cells 1 | NM_016791 | AAAGGAGAGGTCGGACTCGG | AACTGTAGTGTTCTTCCTCGGC | 237 |
| *Traf6* | TNF receptor-associated factor 6 | NM_009424 | ATGCAGAGGAATCACTTGGCA | ACGGACGCAAAGCAAGGTT | 103 |

** Mouse Mmp2 mRNA was not detected using these primers.*

## Supplementary Figures


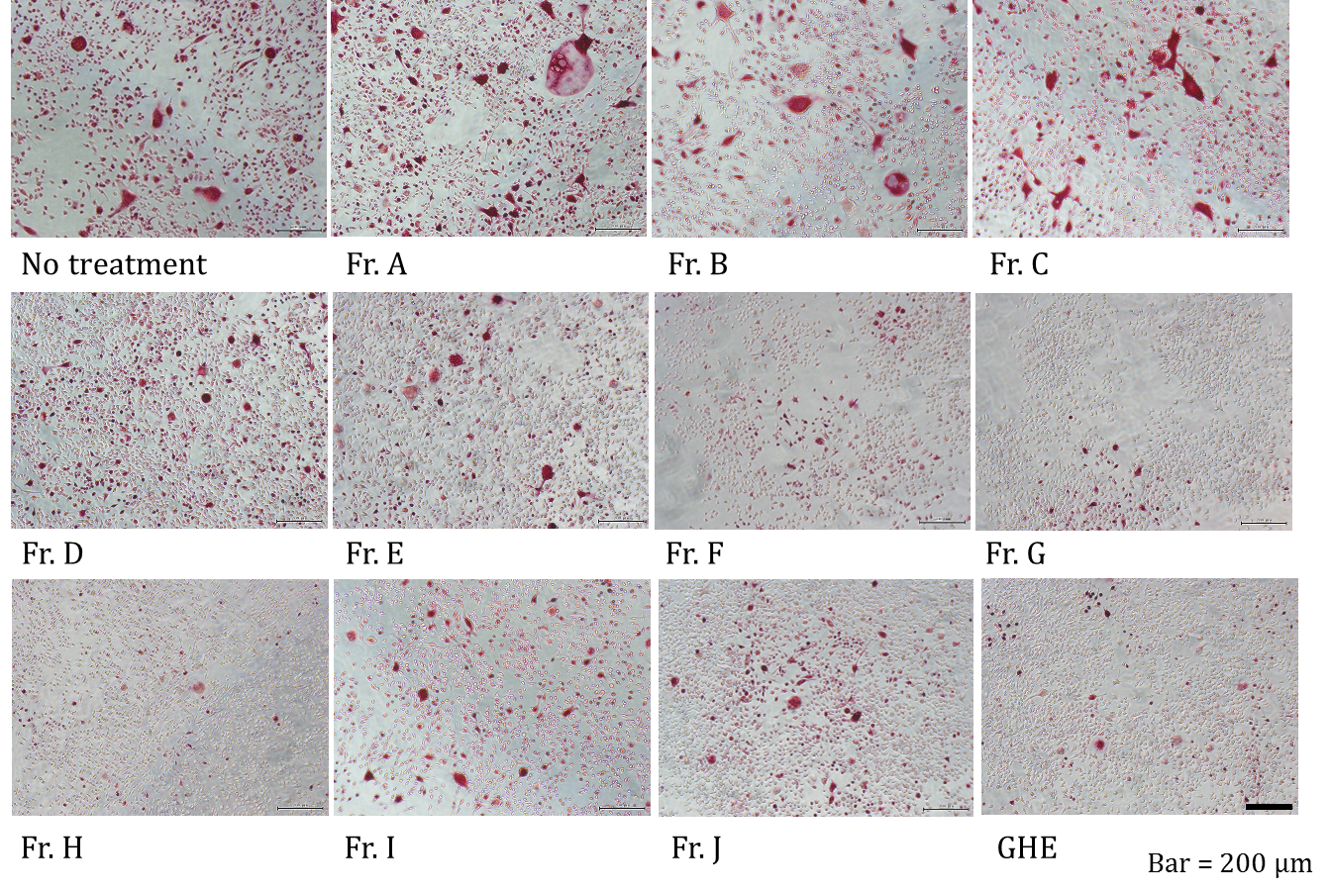


**Supplementary Figure 1.** TRAP staining in RAW264.7 cells treated with GHE or its fractions. Differentiation was induced by sRANKL in RAW264.7 cells with or without GHE (10 µg/mL) or its fractions (20 μg/mL).


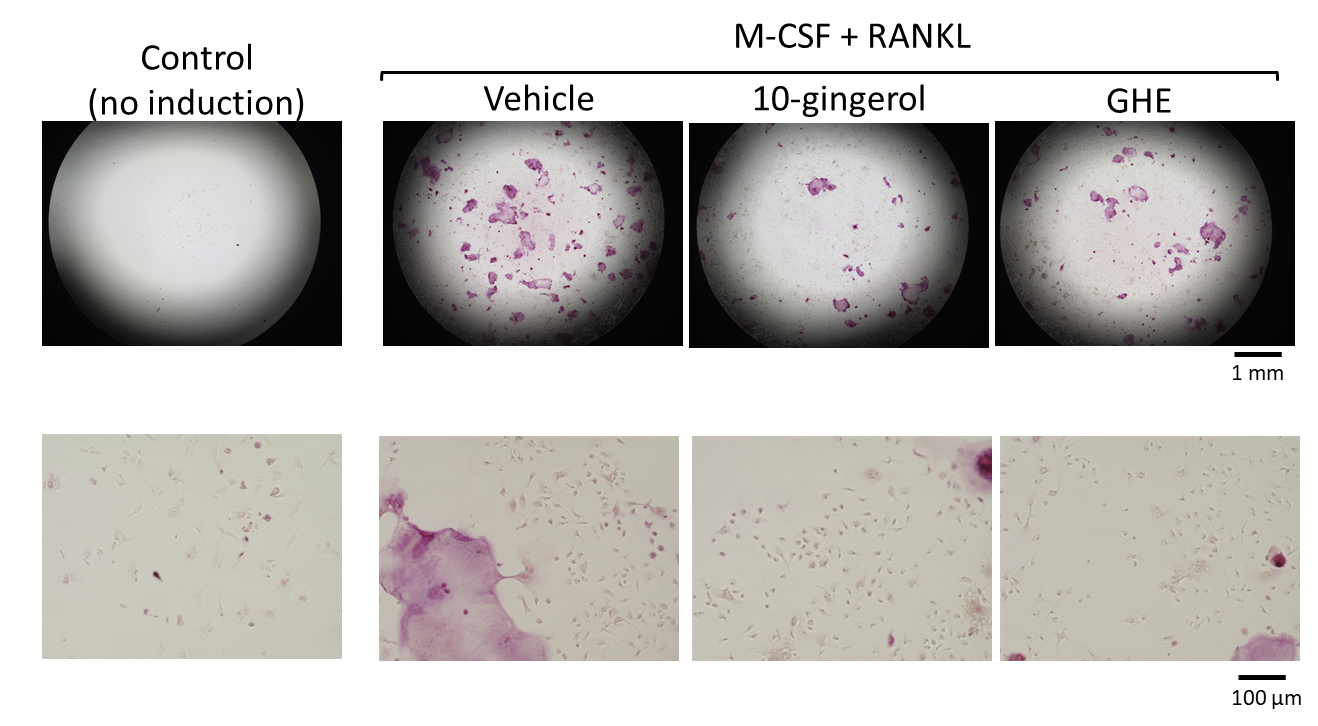


**Supplementary Figure 2.** TRAP staining in rat bone marrow-derived osteoclast precursors treated with 10-gingerol (10 µM) or GHE (10 µg/mL). Differentiation was induced by M-CSF and RANKL with or without GHE (10 µg/mL). Rat bone marrow-derived osteoclast precursors with the differentiation medium were purchased from Cosmo Bio (OSC12C; Tokyo, Japan) and the experiment was performed according to the manufacturer’s protocol. The lower panels show the magnified images.

**Supplementary Figure 3.** Cell viability assay of RAW264.7 cells treated with 6-, 8-, or 10-gingerol. RAW264.7 cells were treated with each gingerol for 48 h and a CellTiter-Glo Cell Viability Assay was performed (Promega, Madison, WI, USA), according to the manufacturer’s instructions. For the positive control, the cells were treated with methotrexate according to a previous study (*PLoS One*. 2013;8:e63073.). The control group was treated with vehicle (0.1% DMSO). n = 8, error bars indicate SE. ns indicates no significant difference.


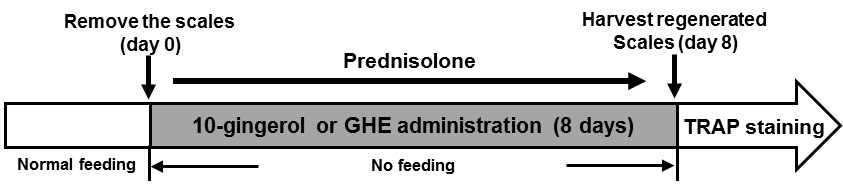


**Supplementary Figure 4.** Experimental design for the zebrafish study. Approximately 30 scales in adult zebrafish were removed (day 0), then prednisolone administration commenced with or without test compounds for eight days to induce osteoporotic regeneration of new scales. On day eight, the regenerated scales were collected and stained by TRAP.

**
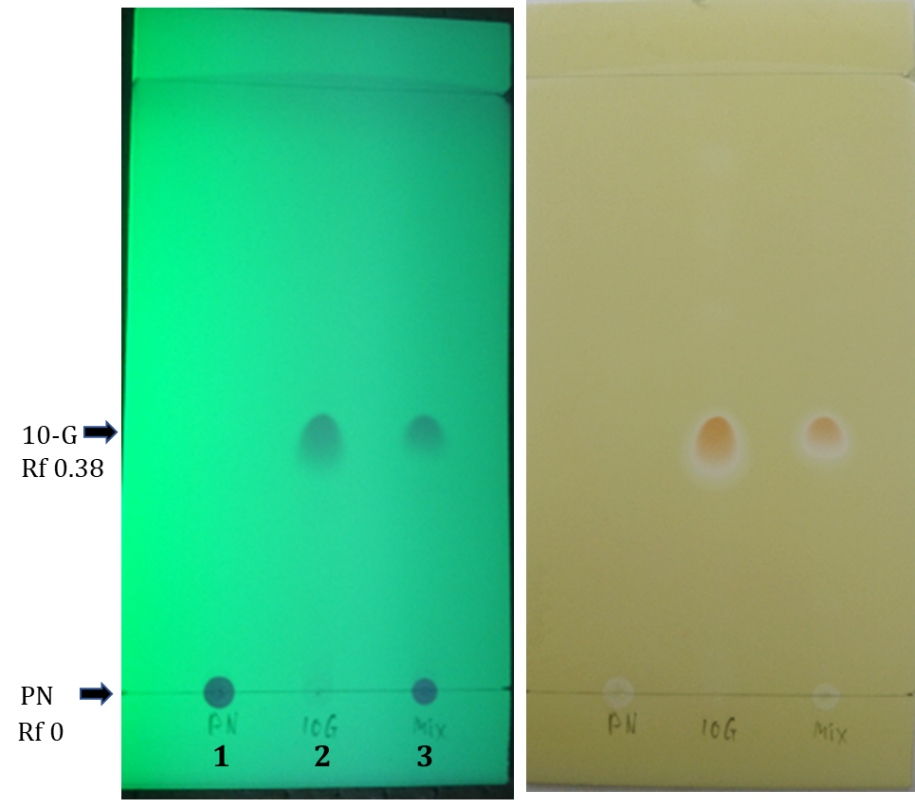
**

**Supplementary Figure 5.** TLC analysis. Prednisolone (PN; lane 1), 10-gingerol (lane 2), and PN with 10-gingerol (lane 3) were loaded on TLC plate and developed with n-hexane:diethyl ether = 2:3 (v/v). After visualization, PN and 10-gingerol were separately detected in lane 3. Right panel: UV light, left panel: 2% Ce(SO_4_)_2_/2N H₂SO₄
